# Microvascular reconstruction of aggressive mandibular desmoplastic fibroma in a child: case report

**DOI:** 10.3389/froh.2026.1752659

**Published:** 2026-03-12

**Authors:** George Batshon, Murad Abdelraziq, Munir Nashashibi, Imad Abu El-Naaj, Yasmin Ghantous

**Affiliations:** 1 Department of Oral and Maxillofacial Surgery, Tzafon Medical Center, Affiliated with Azrieli Faculty of Medicine, Bar Ilan University, Ramat Gan, Israel; 2 Department of Pathology, Tzafon Medical Center, Affiliated with Azrieli Faculty of Medicine, Bar Ilan University, Ramat Gan, Israel

**Keywords:** desmoplastic fibroma, desmoplastic fibroma diagnosis and treatment, fibula free flap reconstruction, microvascular reconstruction, OMFS, OMFS reconstruction, pediatric oncology, segmental mandiblectomy

## Abstract

**Background:**

Desmoplastic fibroma is a rare, benign but locally aggressive fibroblastic tumor of bone, accounting for less than 0.1% of primary bone neoplasms. The mandible is its most frequent site, and occurrence in children is exceptionally uncommon. Because of its infiltrative behavior and the potential for significant functional and aesthetic consequences in growing patients, determining the optimal treatment approach—particularly the role of emerging non-surgical therapies—remains a clinical challenge.

**Case presentation:**

A 10-year-old boy presented with a one-year history of progressive, painless swelling of the right mandible. Imaging revealed an expansive mandibular lesion, and incisional biopsy confirmed desmoplastic fibroma. The family sought evaluation regarding both surgical and non-surgical management options, reflecting current therapeutic uncertainty surrounding this rare tumor in pediatric patients. Following multidisciplinary counseling, definitive surgical treatment was chosen. Using virtual surgical planning and patient-specific 3D-printed guides, a segmental mandibulectomy was performed, followed by immediate reconstruction with a vascularized fibula free flap and a prebent reconstruction plate. Microvascular anastomoses were successful, postoperative imaging demonstrated accurate anatomical restoration, and recovery was uneventful with a stable, well-perfused flap.

**Conclusion:**

This case highlights the diagnostic and therapeutic complexities of pediatric mandibular desmoplastic fibroma and underscores the ongoing debate regarding surgical vs. emerging systemic treatment strategies. Despite increasing interest in non-surgical modalities, wide resection with microvascular reconstruction remains the most reliable approach for achieving durable local control and restoring mandibular continuity in children. Early diagnosis, comprehensive imaging, and advanced virtual surgical planning are essential to optimizing outcomes in these rare and challenging cases.

## Introduction

Desmoplastic fibroma (DF) is an exceedingly rare, benign, yet locally aggressive intraosseous neoplasm that was first described by Jaffe in 1958 as the bone equivalent of desmoid-type fibromatosis (1). Although histologically benign, DF is characterized by its infiltrative growth, bone destruction, and a high propensity for local recurrence following conservative excision (2, 3). It accounts for less than 0.1% of all primary bone tumors and approximately 0.3% of benign osseous lesions (4). Within the maxillofacial skeleton, the mandible is the most commonly affected site, representing up to 22% of all DF cases, typically involving the posterior body, angle, and ramus regions (5, 6).

DF most often presents during the first three decades of life, with a significant proportion occurring in children and adolescents (7). The clinical presentation is usually insidious, with a slowly progressive, painless swelling or facial asymmetry being the most frequent symptom. Despite its benign nature, the lesion's expansile and infiltrative behaviour can lead to cortical perforation, tooth displacement, and soft tissue invasion, occasionally resulting in functional or aesthetic impairment (8). Pain, paraesthesia, or mucosal ulceration are uncommon and typically associated with extensive lesions (9).

Radiographically, DF appears as a radiolucent lesion, either unilocular or multilocular, with poorly defined or scalloped borders, cortical expansion, and thinning or perforation (10). Computed tomography (CT) further delineates the lesion's relationship to cortical plates and adjacent structures, while magnetic resonance imaging (MRI) may demonstrate soft tissue involvement and internal signal heterogeneity, particularly in aggressive variants (11). These radiologic characteristics, however, are nonspecific and can mimic other odontogenic and non-odontogenic lesions such as ameloblastoma, odontogenic myxoma, fibrous dysplasia, or central giant cell granuloma (12).

Histopathologically, DF is composed of uniform spindle-shaped fibroblasts arranged in interlacing fascicles within a dense collagenous stroma (13). Cellular atypia and mitotic figures are rare, supporting its benign histology, though its infiltrative margins make complete surgical excision essential. Immunohistochemical findings are variable; many lesions demonstrate cytoplasmic or, less frequently, nuclear β-catenin positivity, reflecting partial activation of the Wnt signalling pathway (14). DF typically stains positive for vimentin and variably for smooth muscle actin (SMA), while being negative for desmin, S-100, and cytokeratins—features that help distinguish it from myo-fibroblastic or neural tumors (15). Recent molecular studies suggest that DF of the jaw may have a distinct developmental origin compared to desmoid-type fibromatosis, possibly due to the neural crest-derived embryology of craniofacial bones (16).

The treatment of DF remains primarily surgical. Simple curettage or enucleation has been associated with recurrence rates as high as 40%–70%, whereas wide resection with histologically clear margins significantly reduces this risk (3, 7, 10). However, in pediatric patients, the extent of resection must balance oncologic control with preservation of mandibular growth, occlusion, and function. Reconstruction of large mandibular defects in this context poses a major challenge. Vascularized bone grafts, particularly the fibula free flap, are now regarded as the gold standard for immediate reconstruction following segmental mandibulectomy, offering structural integrity, revascularization potential, and long-term adaptability for dental rehabilitation and craniofacial growth (5, 9).

The rarity of DF, especially in the pediatric population, limits the availability of robust clinical data, with most reports being isolated case studies or small series. Nevertheless, the accumulating literature consistently emphasizes the importance of multidisciplinary management—integrating surgical oncology, pediatric maxillofacial surgery, and reconstructive microvascular expertise—to achieve both oncologic clearance and functional restoration.

Herein, we present the case of a 10-year-old boy with a large desmoplastic fibroma of the right mandible extending from the parasymphyseal region to the ascending ramus. The patient was treated with segmental mandibulectomy and immediate microvascular reconstruction using a 3D-planned fibula free flap. This case highlights the diagnostic complexity, therapeutic decision-making considerations, and the advanced surgical and reconstructive planning required for managing an extensive pediatric mandibular desmoplastic fibroma.

## Case presentation

A healthy 10-year-old male presented to the Oral and Maxillofacial Surgery (OMFS) department with a one-year history of progressive swelling in the right mandible. The swelling was painless, and the child reported no systemic symptoms or functional limitations. His medical and family histories were unremarkable. Notably, the patient had initially been evaluated at another hospital, where the diagnostic workup was initiated before the family sought further assessment and management at our institution.

## Clinical examination

**Figure 1:**
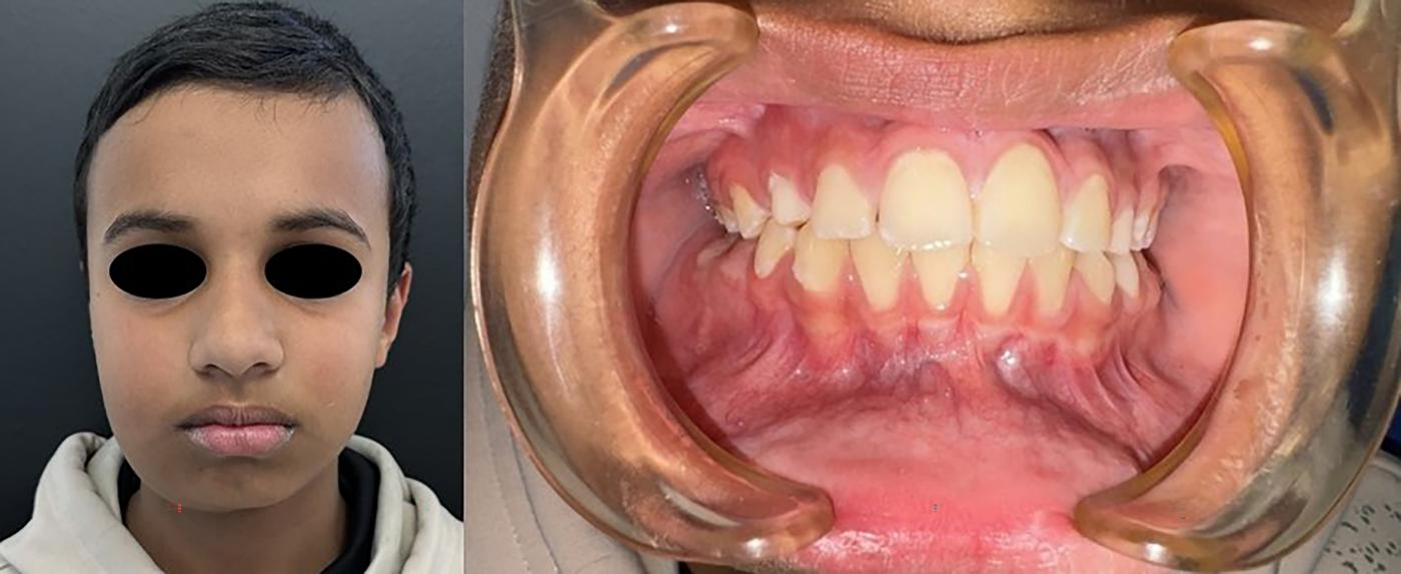
Initial clinical presentation of the patient. Left: Extraoral photograph demonstrating a firm, non-tender swelling along the right mandibular border without erythema or facial asymmetry at rest. Right: Intraoral photograph showing intact mucosa, normal tongue mobility, and no visible soft-tissue abnormalities, despite the underlying expansile mandibular lesion.

Extraoral examination revealed a firm, non-tender swelling along the right mandibular border without erythema, warmth, or fluctuance (Figure 1). There was no facial nerve weakness or cervical lymphadenopathy. Mouth opening was normal. Intraoral evaluation demonstrated intact mucosa, normal tongue mobility, and no elevation of the floor of the mouth. No dental or soft tissue abnormalities were present (Figure 1). Routine laboratory tests—including complete blood count, chemistry panel, and coagulation profile—were within normal limits.

## Imaging studies

### Panoramic radiograph (OPG)

**Figure 2:**
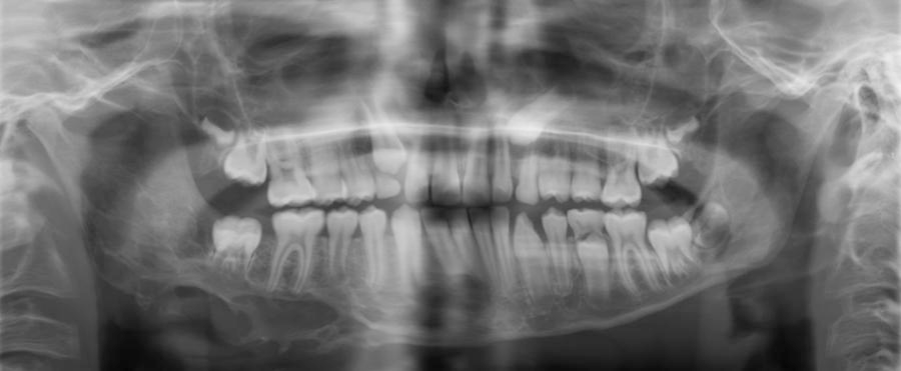
OPG demonstrating the mandibular lesion. The OPG reveals a large, ill-defined radiolucent lesion extending from the right para-symphyseal region across the right mandibular body and retromolar area to the ascending ramus.

The OPG demonstrated a large, poorly defined radiolucent lesion extending from the left parasymphyseal region through the right mandibular body and retromolar area to the ascending ramus (Figure 2).

### Computed tomography (CT)

A high-resolution maxillofacial CT scan was obtained to further characterize the lesion's bony involvement and anatomical extent. Axial, coronal, and sagittal reconstructions demonstrated a large, expansile, osteolytic lesion involving the mandible from the left parasymphyseal region through the right mandibular body and extending posteriorly to the ascending ramus.

**Figure 3:**
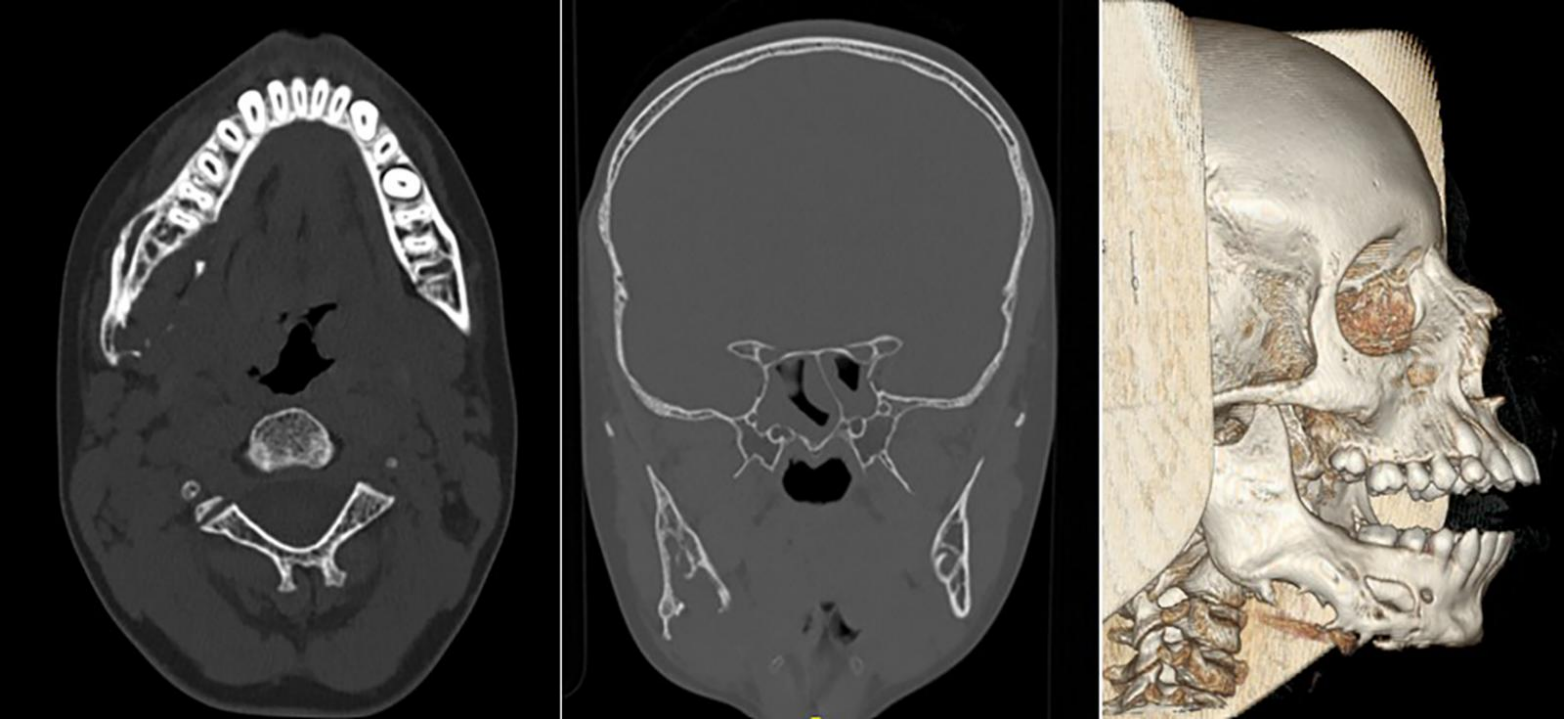
Computed tomography (CT) evaluation of the mandibular lesion. Left: Axial CT image showing an expansile osteolytic lesion of the right mandible with cortical thinning and focal lingual perforation. Middle: Coronal CT image demonstrating vertical extension of the lesion, replacement of normal trabecular bone, and involvement from the para-symphysis to the ascending ramus. Right: 3D reconstructed CT view highlighting the extent of mandibular destruction and external cortical deformation caused by the lesion.

The borders of the lesion were predominantly ill-defined, with areas of cortical thinning, expansion, and focal breakthrough. Marked lingual cortical perforation was evident along the right mandibular body. The buccal cortex was also significantly thinned, and a site of cortical discontinuity corresponded with the clinically observed buccal perforation (Figure 3).

### Magnetic resonance imaging (MRI)

MRI of the head and neck was performed using T1, T2, fat-suppressed, and contrast-enhanced sequences. Imaging revealed a homogeneous soft-tissue mass replacing a large portion of the mandible on the right side. The lesion measured approximately 36 mm craniocaudally, 40 mm transversely, and 48 mm sagittally. It extended into surrounding soft tissues but spared the condylar head. No regional lymphadenopathy was identified, and salivary glands appeared normal (Supplementary Figure S1).

## Diagnosis

An incisional biopsy performed at the referring hospital confirmed the diagnosis of desmoplastic fibroma, a benign but locally aggressive fibroblastic tumor of bone. At that institution, systemic therapy with pazopanib was proposed as a non-surgical management option. However, given the limited evidence supporting targeted medical therapy for desmoplastic fibroma—particularly in pediatric osseous lesions—and the potential toxicity associated with such treatment in children, the family sought further evaluation. After comprehensive multidisciplinary counseling at our center, they elected to proceed with definitive surgical management.

## Surgical management

Following confirmation of the diagnosis, multidisciplinary evaluation focused on determining the most appropriate reconstructive strategy given the extent of mandibular destruction. The extensive segmental destruction of the mandibular body and ramus required a reconstructive modality that could reliably reconstitute a long, structurally robust bony segment while supporting functional rehabilitation and pediatric craniofacial development. A fibula free flap was therefore selected as the preferred reconstructive option. Preoperative planning included high-resolution 3D CT imaging of the head and neck, which enabled precise virtual surgical planning and the fabrication of patient-specific cutting guides and a pre-bent reconstruction plate (Supplementary Figures S2–S4). CT angiography of the lower extremities confirmed suitable vascular anatomy for safe fibula flap harvest.

## Mandibular resection

Under general anesthesia with nasal intubation, a cervical apron incision was used to expose the mandible. The lesion's buccal cortical perforation was visualized. Custom 3D-printed cutting guides were fixed to the mandibular symphysis and sigmoid notch. Guided osteotomies were performed, and the tumor-bearing mandibular segment was removed. Drill holes for plate fixation were pre-established according to the virtual plan. A pre-bent titanium reconstruction plate was secured to the residual mandible with bi-cortical screws.

## Fibula free flap reconstruction

Simultaneously, a left fibula free flap was harvested. A lateral leg incision exposed the fibula while preserving the superficial peroneal nerve and perforators to the skin paddle. Proximal and distal osteotomies were completed, and the vascular pedicle was preserved until flap division initiated the ischemia time. The donor site was closed in layers with placement of a JP-7 drain. The fibula was segmented and shaped according to the 3D virtual surgical plan and fixed to the reconstruction plate using mono-cortical screws.

## Microvascular anastomosis and closure

Microvascular anastomoses were performed between the fibula flap pedicle and the right facial artery and external jugular vein under an operating microscope using 9–0 nylon sutures. Adequate venous flow was confirmed intraoperatively with Doppler probes. The neck was closed in layers, and two JP drains were placed.

## Postoperative course

**Figure 4:**
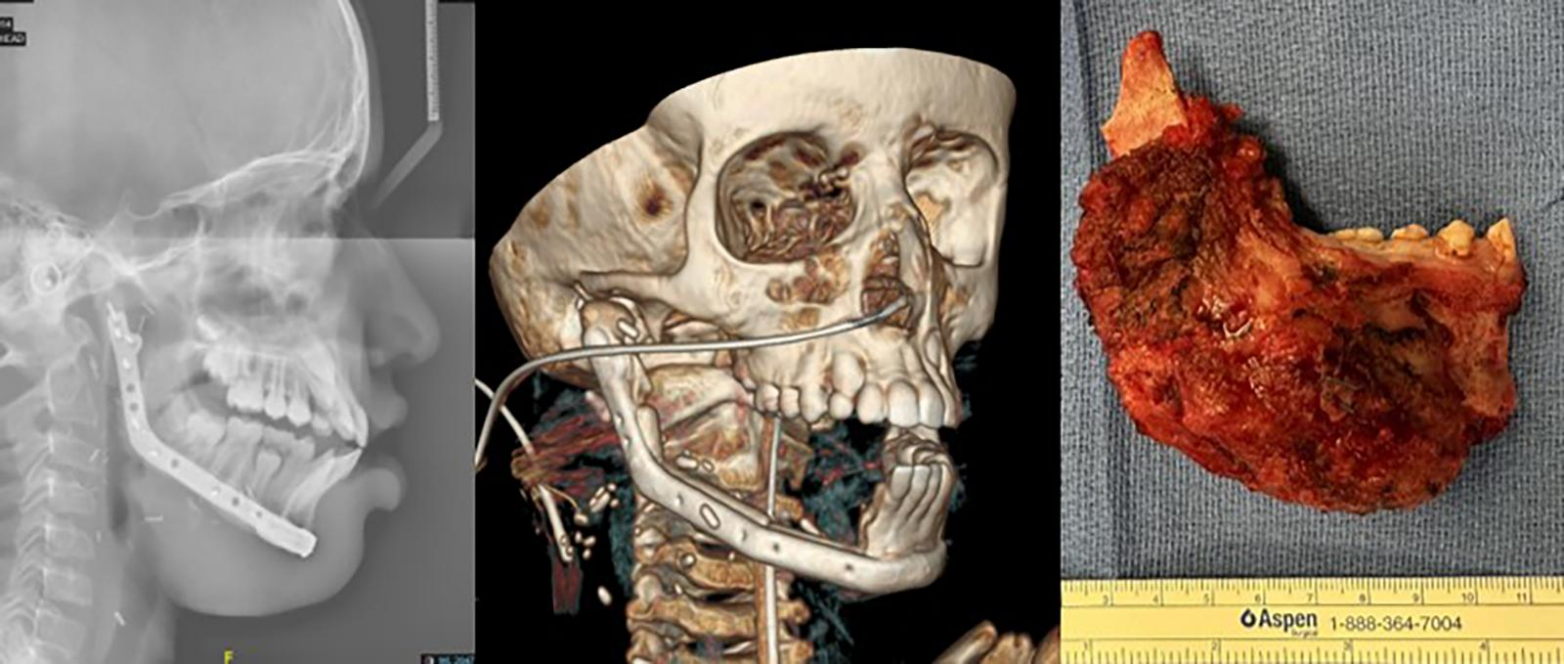
Postoperative radiographic evaluation following mandibular reconstruction and resected mandibular specimen. Left: Lateral cephalometric radiograph demonstrating proper alignment of the reconstructed mandible. Middle: 3D CT reconstruction illustrating the final mandibular contour achieved with the fibula free flap and prebent reconstruction plate. Right: Intraoperative photograph of the en-bloc resection specimen, demonstrating the removed segment of mandible with the entire tumor mass included.

The patient was transferred intubated and sedated to the pediatric ICU for postoperative monitoring. Postoperative CT scans and radiographs confirmed accurate alignment of the fibula segments and stability of the reconstruction. The reconstruction closely matched the 3D surgical plan and provided excellent early functional and aesthetic outcomes (Figure 4). The full resection specimen was processed for definitive histopathological examination. Microscopy revealed uniform spindle-shaped fibroblasts arranged in fascicles within a dense collagenous matrix, exhibiting minimal atypia and rare mitotic figures, thereby confirming the diagnosis of desmoplastic fibroma (Supplementary Figure S5).

## Radiological and clinical follow-up

At 15-month follow-up, panoramic radiography demonstrated stable integration of the fibula graft with satisfactory bony continuity and maintenance of mandibular contour, without evidence of recurrence or hardware-related complications (Supplementary Figure S6). Additionally, imaging revealed satisfactory growth of the right mandibular ramus with appropriate elongation of the condylar complex, indicating preservation of the right condylar growth center. The reconstructed condylar–ramus unit exhibited excellent function, comparable to that of a native temporomandibular joint (Supplementary Figure S6).

Clinically, the patient exhibited satisfactory facial symmetry (Supplementary Figure S7, left), normal mandibular range of motion without limitation in mouth opening or temporomandibular joint mobility, stable occlusion, healthy mucosa with stable occlusion and preserved oral function (Supplementary Figure S7, right). After postoperative recovery, the patient was referred for orthodontic evaluation and management to prevent overeruption of the right maxillary teeth. This was necessitated by removal of the opposing mandibular dentition during segmental mandibulectomy, with the goal of maintaining vertical dimension and facilitating future implant-supported rehabilitation and occlusal stability. The current orthodontic treatment plan consists of daily wear of maxillary and mandibular Invisalign aligners.

## Discussion

Desmoplastic fibroma (DF) of the mandible is an uncommon benign but locally aggressive fibroblastic neoplasm that presents unique diagnostic and therapeutic challenges, particularly in pediatric patients (7, 10, 15–18). Review of the pediatric literature demonstrates a marked mandibular predominance of desmoplastic fibroma, with approximately 94% of reported pediatric jaw cases involving the mandible (1–18). Management has most commonly consisted of local excision or curettage, frequently combined with non-vascularized reconstruction or staged augmentation, while segmental mandibulectomy has generally been reserved for extensive or recurrent disease (1–18). Most authors favoured rib or costochondral grafts, curettage or enucleation with bone grafting, or delayed reconstruction, often citing concerns related to young patient age, ongoing craniofacial growth, and donor-site morbidity. Microvascular reconstruction using a free fibula flap appears to be rarely reported in children with mandibular desmoplastic fibroma, reflecting both the exceptional nature of such cases and the absence of consensus regarding optimal reconstructive strategies in growing patients (9). These findings underscore the rarity of extensive pediatric mandibular DF requiring free flap reconstruction and highlight the uniqueness of the present case, emphasizing the importance of individualized treatment planning, careful preservation of growth centers, and long-term follow-up. The rarity of this tumor within the craniofacial skeleton and the absence of pathognomonic clinical features often contribute to delayed diagnosis. In children, DF may initially be mistaken for odontogenic, inflammatory, or developmental conditions, emphasizing the importance of thorough radiographic and histopathological evaluation (2, 6, 7, 18–23). In the present case, the patient exhibited a painless, slowly progressive swelling of the mandible—consistent with the generally indolent yet infiltrative nature of DF—which was ultimately confirmed via incisional biopsy.

Advanced imaging played a central role in defining the extent of disease. The lesion demonstrated an expansive and destructive growth pattern with cortical perforation and soft-tissue extension, findings that have been described in multiple isolated case reports of mandibular DF in children (6, 7, 10–13, 19, 20). Although the radiographic appearance of DF is variable, several authors have highlighted its capacity for cortical thinning, perforation, and medullary infiltration (6, 7, 10, 12, 15, 16, 18–20). The combination of CT and MRI in our case enabled precise visualization of bony destruction, medullary involvement, and soft-tissue spread—information essential for surgical planning in growing patients. Three-dimensional imaging further contributed to preoperative accuracy through virtual planning, custom cutting guides, and pre-bent fixation plates, building on principles increasingly used in complex maxillofacial reconstruction.

From a diagnostic standpoint, DF remains a mimicker of a wide range of odontogenic and non-odontogenic lesions, including ameloblastoma, odontogenic myxoma, central giant cell granuloma, and fibrous dysplasia (2, 7, 10, 15, 16, 18, 20, 21). Histopathologically, our case demonstrated the classic appearance of DF: uniform spindle cells arranged in fascicles within a collagen-rich stroma, with minimal atypia and low mitotic activity, in keeping with previously reported mandibular and gnathic cases (7, 10, 15, 16, 18, 20–22). Immunohistochemical and molecular findings have been variably reported. Kadowaki et al. identified a CTNNB1 point mutation with nuclear β-catenin accumulation in a mandibular DF, suggesting a role for Wnt/β-catenin signalling in at least some cases (1). In contrast, other series of jaw DF have found heterogeneous or absent nuclear β-catenin staining, indicating potential biological diversity among these tumors (15, 16). These observations emphasize that while ancillary studies may support diagnosis in selected cases, DF remains primarily a morphologic and clinical-radiologic diagnosis, and surgical management continues to be guided by conventional histology and imaging rather than molecular profiling alone.

Surgery remains the cornerstone of DF management because of its infiltrative behavior and risk of local recurrence (7, 10, 15, 16, 18). Various surgical approaches have been described, ranging from curettage and enucleation to marginal and segmental resection (6, 7, 10–12, 16, 18–22). The choice of treatment must be tailored to the lesion's size, anatomical involvement, and patient age. Limited excision may preserve surrounding structures but has been associated with residual or recurrent disease, particularly when radiologic and histologic margins are ill-defined (7, 10, 16, 18). In contrast, wider resection has been favored in more extensive or aggressive lesions to reduce the likelihood of persistent microscopic tumor (7, 10, 11, 15, 16, 18).

In the present case, the lesion's extensive destruction of the mandibular body and ramus, along with cortical perforation and soft-tissue extension, necessitated segmental mandibulectomy—a treatment approach also reported in other severe pediatric DF cases (7, 9–11, 15, 16, 18–20, 24). Segmental resection was selected to ensure complete disease clearance while minimizing recurrence risk. Although DF does not metastasize, its potential for local recurrence justifies meticulous resection and close postoperative follow-up (7, 10, 15, 16, 18).

Reconstruction of mandibular defects in pediatric patients presents unique challenges due to ongoing craniofacial growth and the need to restore symmetry, occlusion, and long-term function. Although vascularized fibula grafts lack intrinsic growth potential comparable to native mandibular bone, the fibula free flap has emerged as a robust option for long-segment mandibular defects, providing reliable restoration of continuity, contour, and potential for future dental rehabilitation (8, 9). Several pediatric series have demonstrated that early restoration of mandibular continuity using fibula free flaps offers a stable scaffold for craniofacial development, with acceptable functional and aesthetic outcomes when combined with long-term surveillance and staged interventions as needed (9).

Skinner et al (9). reported successful mandibular reconstruction with fibula free flap in a pediatric desmoplastic fibroma case, with maintained function and symmetry at six-year follow-up, while emphasizing the necessity for prolonged monitoring throughout growth. Similarly, Ferri et al (11). highlighted the importance of balancing oncologic clearance with preservation of craniofacial development in children with mandibular desmoplastic fibroma, noting that aggressive resection may be required but must be accompanied by careful reconstructive planning and extended follow-up. Woods et al (7). further underscored that although wide resection reduces recurrence risk, pediatric patients frequently require long-term multidisciplinary care to address evolving occlusal and facial growth changes.

Additionally, findings by Thuau et al. (25) in pediatric tibial reconstruction reinforce that vascularized fibula grafts, while mechanically stable, do not adapt to skeletal growth in the same manner as native bone. Their report emphasizes the importance of monitoring growth-related alignment or volume discrepancies over time and supports staged approaches when planning definitive functional or prosthetic rehabilitation (25). This broader pediatric reconstructive principle informs our own management and surveillance strategy.

In our case, virtual surgical planning enabled precise adaptation of the fibula graft to the defect, ensuring accurate three-dimensional reconstruction. The fibula provided structural stability, a favorable cortical profile, and compatibility with a pre-bent reconstruction plate, thereby supporting both early function and long-term craniofacial development while mitigating risks of facial asymmetry and occlusal discrepancies.

The microsurgical aspect of treatment warrants emphasis. Pediatric microvascular reconstruction can be challenging due to smaller vessel calibers and demanding postoperative monitoring, yet successful outcomes have been consistently reported in mandibular DF using fibula free flaps (8, 9). In our patient, microvascular anastomoses to the facial artery and external jugular vein were achieved without complication, and serial Doppler assessment confirmed flap viability. This experience aligns with pediatric cases previously described, in which vascularized bone reconstruction provided stable and functional outcomes in large mandibular defects (8, 9, 11, 17).

Although a double-barrel fibula flap was considered to enhance vertical bone height for future implant rehabilitation, this approach was ultimately avoided. The patient's young age and the approximately 12 cm mandibular defect prompted prioritization of flap reliability and surgical safety, leading to selection of positioning the fibula along the inferior mandibular border. In pediatric patients, double-barrel reconstruction may introduce unnecessary risk, including ischemic compromise related to periosteal disruption and distal segment perfusion, excessive graft bulk, and susceptibility to venous compromise from vessel twisting or compression. Reported complications include infection, hardware exposure, vascular thrombosis, bone resorption, and occasional flap loss (26–29). Furthermore, the largest pediatric series to date demonstrated substantial morbidity associated with double-barreled vascularized fibula reconstruction, with revision surgery required in 75% of patients, pseudarthrosis in 50% (predominantly septic), and limb loss in 12.5%, underscoring the vulnerability of this technique in growing patients despite acceptable consolidation in successful cases (25). As an alternative strategy, staged anterior iliac crest bone grafting is planned following skeletal maturity to augment vertical bone height and facilitate prosthetic rehabilitation. This staged approach is consistent with current pediatric reconstruction paradigms, offering increased flexibility and safety while minimizing early operative risk, and supports delayed augmentation as a reliable pathway toward functional dental rehabilitation in growing children (26–29).

Comparisons with previously published pediatric DF cases highlight several important considerations. DF has been reported across a broad pediatric age range, most commonly presenting as painless swelling, facial asymmetry, or functional disturbances such as trismus or malocclusion (6–8, 10–13, 18–22, 24). Surgical approaches in those reports have ranged from conservative curettage for small, well-contained lesions to wide or segmental resection for extensive disease (6, 7, 10–12, 16, 18–22). Our patient's presentation—marked by cortical perforation and significant soft-tissue extension—mirrors the more aggressive cases described in the literature, supporting the decision for segmental resection rather than conservative treatment.

Reconstruction strategies have varied as well. While some authors favor non-vascularized rib grafts for selected pediatric defects (10, 11), others highlight the superiority of vascularized fibula flaps for large or complex reconstructions due to their length, bone stock, and compatibility with osseo-integrated implants (8, 9). In the present case, virtual planning facilitated accurate restoration of the mandibular contour and condylar position, critical for promoting symmetrical facial growth in a child.

Long-term follow-up is crucial, particularly because recurrence after conservative surgery has been reported in individual jaw cases (7, 10, 16, 18–22). More extensive resections generally correlate with improved local control, though data remain limited. Our patient, who is now approximately 15 months postoperatively and shown no evidence of recurrence to date, will therefore undergo long-term clinical and radiologic surveillance, extending into adulthood due to DF's slow-growing nature and the long duration of craniofacial development.

This case also highlights the importance of multidisciplinary care, involving pediatric maxillofacial surgeons, microvascular reconstructive surgeons, radiologists, anesthesiologists, and pathologists. Similar comprehensive management has been emphasized in other complex pediatric DF cases (7–11, 15–18), contributing to optimal outcomes. In our case, multidisciplinary coordination enabled seamless integration of virtual planning, oncologic resection, and immediate microvascular reconstruction.

Although wide surgical resection remains the only consistently effective therapy for DF of the jaws, several non-surgical and adjuvant treatments have been described, though evidence remains extremely limited. Conservative curettage has been associated with high recurrence rates (7, 10, 16, 18), whereas radiotherapy is used only in exceptional circumstances due to long-term risks in children (10, 16–18). Pharmacologic therapies—including NSAIDs, anti-estrogen agents, and low-dose regimens such as methotrexate and vinblastine—are extrapolated from desmoid-type fibromatosis but lack validation in DF. More recently, targeted agents such as tyrosine kinase inhibitors have gained interest. Lenvatinib, alone or combined with denosumab, has shown activity in preclinical DF models, though no clinical cases treated with pazopanib or similar agents have been reported.

Although pazopanib has not been used clinically in desmoplastic fibroma of the jaws, its potential relevance derives from its established activity in desmoid-type fibromatosis, a biologically related fibroblastic neoplasm. Pazopanib is an oral multi-targeted tyrosine kinase inhibitor (TKI) that blocks VEGFR-1/2/3, PDGFR-α/β, FGFR-1/3, and c-KIT, thereby inhibiting tumor angiogenesis and stromal proliferation (30). These mechanisms are particularly effective in desmoid tumors, where aberrant Wnt/β-catenin signalling and hyper-vascular stromal proliferation contribute to tumor growth. Clinical studies have demonstrated meaningful responses to pazopanib in patients with aggressive fibromatosis, including symptomatic improvement and reduction in tumor size (31). Desmoplastic fibroma shares several histopathologic and molecular features with desmoid tumors—most notably uniform fibroblastic proliferation within a collagen-rich stroma and, in some cases, alterations in CTNNB1/β-catenin—yet important differences remain. Unlike desmoid tumors, DF is a primary bone tumor, typically less vascular and more structurally infiltrative. These differences partly explain why no clinical cases of DF treated with pazopanib, sorafenib, or other TKIs have been reported. The only experimental evidence of TKI efficacy in DF comes from the recent work of De Vita and colleagues, who demonstrated significant inhibitory effects of Lenvatinib, particularly when combined with denosumab, in patient-derived DF cultures and a zebrafish xenograft model (24). This highlights the theoretical—but unproven—potential for angiogenesis- or TKI-targeted therapy in unresectable DF. Importantly, pazopanib carries well-documented toxicity in pediatric populations, including hypertension, hepatotoxicity, impaired wound healing, hypothyroidism, proteinuria, and cardiotoxicity, as demonstrated in pediatric oncology trials (32). These risks are particularly relevant in children requiring maxillofacial reconstruction or dental rehabilitation. Therefore, although pazopanib represents a mechanistically appealing agent based on parallels with desmoid tumors, its use in DF should currently be considered experimental and inappropriate as first-line therapy (32). Surgical resection remains the gold standard for durable disease control in mandibular DF.

Given the rarity of desmoplastic fibroma in the pediatric population and the heterogeneity of reported cases, there is a clear need for a comprehensive systematic review focused specifically on children. Existing literature consists almost entirely of isolated case reports and small series, which limits the ability to identify consistent clinical patterns, radiographic characteristics, recurrence risks, and age-specific treatment considerations. A dedicated pediatric systematic review would allow for more robust comparison of surgical and non-surgical modalities, clarify the effectiveness and limitations of emerging therapies such as targeted agents, and help refine evidence-based recommendations for diagnosis, resection margins, reconstructive planning, and follow-up. Such an effort would provide valuable insights into the behavior of this tumor in growing patients and support more informed clinical decision-making in future cases.

When comparing surgical and non-surgical approaches for desmoplastic fibroma of the mandible, the distinction remains clear. Non-surgical therapies lack robust evidence and can be considered only in rare circumstances of unresectable disease or when medical comorbidities preclude operative intervention. In contrast, surgical excision—particularly wide or segmental resection—continues to represent the gold standard for achieving durable local control. Contemporary reconstructive strategies, including virtual surgical planning and vascularized fibula free flaps, have markedly improved the precision and predictability of mandibular reconstruction. These advances allow for accurate restoration of mandibular form, occlusion, and facial symmetry, while minimizing the functional morbidity traditionally associated with extensive resection. The successful functional and aesthetic outcomes observed in our patient further support the value of 3D-planned fibula reconstruction in extensive pediatric DF.

In summary, this case underscores the complex challenges inherent in diagnosing and managing desmoplastic fibroma of the mandible in children, particularly when lesions demonstrate significant bony destruction and soft-tissue extension. Our experience highlights the necessity of early recognition, comprehensive imaging, and multidisciplinary evaluation in guiding optimal treatment selection. Although emerging systemic therapies have generated interest, surgical resection supported by advanced virtual planning and microvascular reconstruction remains the most reliable and effective approach for long-term disease control in pediatric patients. Continued accumulation of high-quality, well-documented pediatric cases with extended follow-up will be essential to refine therapeutic strategies, enhance understanding of DF biology in growing patients, and ultimately support the development of clearer evidence-based clinical guidelines for this exceptionally rare tumor.

## Data Availability

The raw data supporting the conclusions of this article will be made available by the authors, without undue reservation.
