# Supplementary material for: Microvascular reconstruction of aggressive mandibular desmoplastic fibroma in a child: case report
Source: Front Oral Health. 2026 Mar 12;7:1752659. doi: 10.3389/froh.2026.1752659 (PMC13018102; doi:10.3389/froh.2026.1752659)
Supplement: Supplementary file 1 [file Datasheet2.docx]

**Microvascular Reconstruction of Aggressive Mandibular Desmoplastic Fibroma in a Child: Case Report**


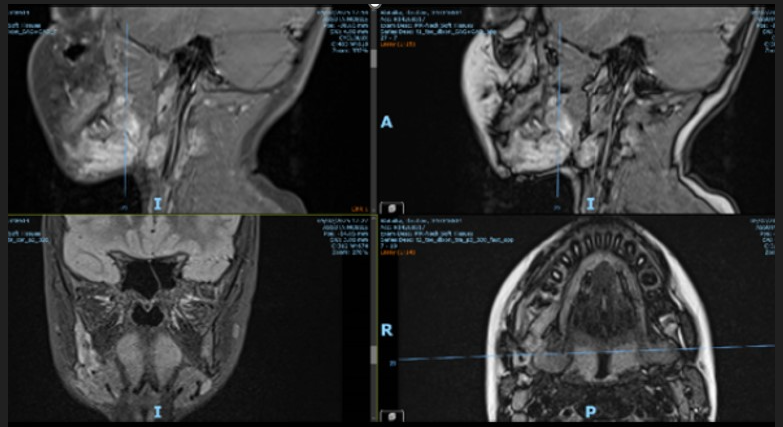
**Supplemental Figures**

**Supplementary Figure 1. Preoperative MRI of the mandible demonstrating extent of desmoplastic fibroma.** Multiplanar MRI sequences (axial, coronal, and sagittal views) showing a large, homogeneously enhancing soft-tissue mass replacing the right mandibular body and ramus.


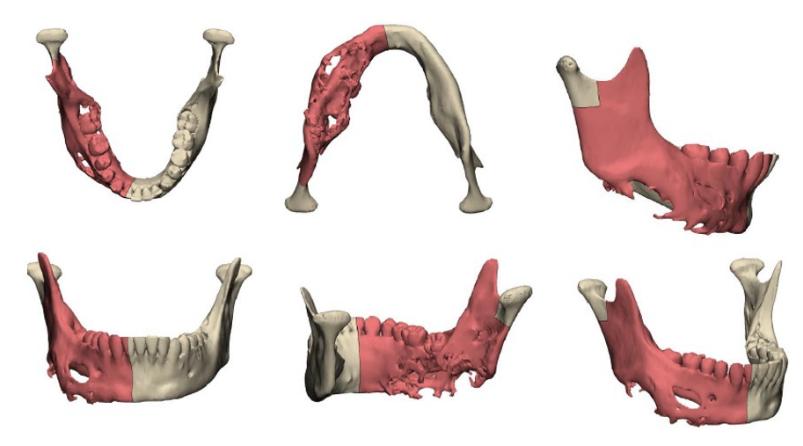


**Supplementary Figure 2. Three-dimensional virtual surgical planning of mandibular tumor resection.** 3D reconstructed mandibular models illustrating the planned segmental resection for desmoplastic fibroma. The affected portions of the mandible are highlighted in red, delineating the full extent of osseous involvement as determined by CT and MRI. Multiple views (anterior, superior, lateral, and oblique) demonstrate the planned osteotomy lines, the boundaries of tumor removal, and the anticipated defect configuration. This virtual planning guided the fabrication of patient-specific cutting guides and informed precise intraoperative execution of the segmental mandibulectomy.


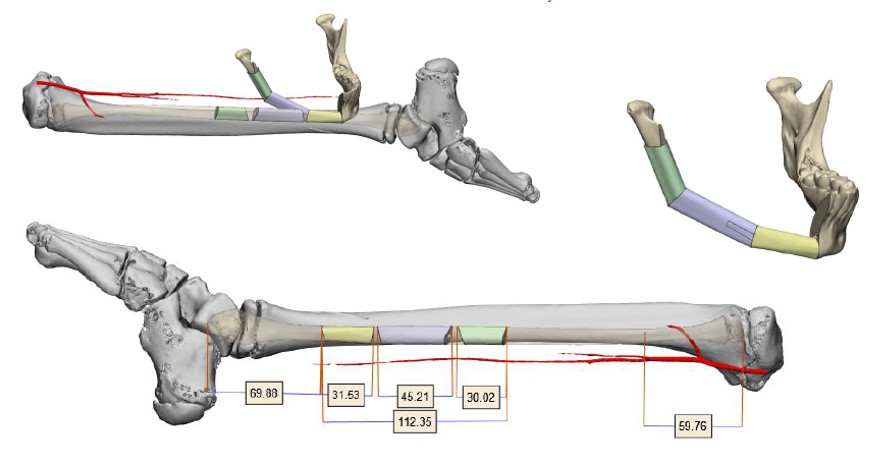


**Supplementary Figure 3. Virtual planning of fibula osteotomies for mandibular reconstruction.** Three-dimensional virtual surgical planning illustrating the design of segmented fibula osteotomies for reconstruction of the mandibular defect. The fibula is shown with marked osteotomy sites and segment lengths, corresponding to the dimensions required to recreate the mandibular contour. Color-coded fibula segments are aligned to the planned mandibular reconstruction, demonstrating precise adaptation to the neomandible shape. The course of the peroneal vascular pedicle (red) is visualized to ensure preservation of perfusion during osteotomy planning. This virtual workflow enabled accurate intraoperative fibula shaping and optimized the fit of the fibula segments to the patient-specific prebent reconstruction plate.

**
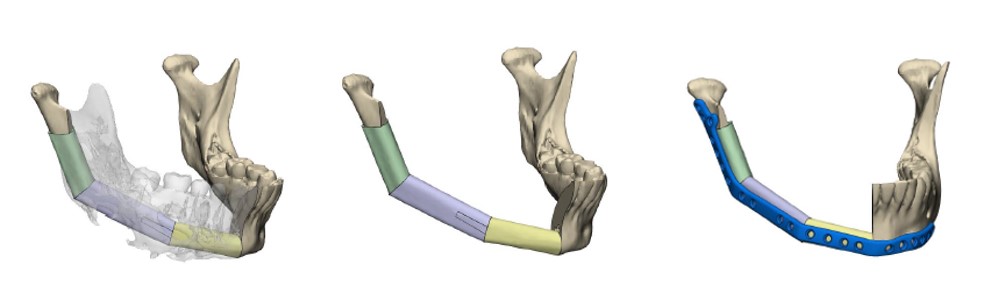
**

**Supplementary Figure 4. Virtual alignment of segmented fibula graft with the mandibular defect and pre-bent reconstruction plate.** Sequential 3D renderings demonstrating the virtual reconstruction workflow.
Left: Initial positioning of the color-coded fibula segments against the mirrored mandibular template, ensuring accurate reproduction of the native contour.
Middle: Refined alignment of fibula segments forming the neo-mandible after virtual adjustment of angulation and segment length.
Right: Final reconstruction plan showing the fibula segments secured to the patient-specific pre-bent reconstruction plate, designed to restore mandibular continuity and symmetry.
This virtual planning process enabled precise intraoperative adaptation of the fibula graft and optimized fixation using a custom pre-bent plate.

**
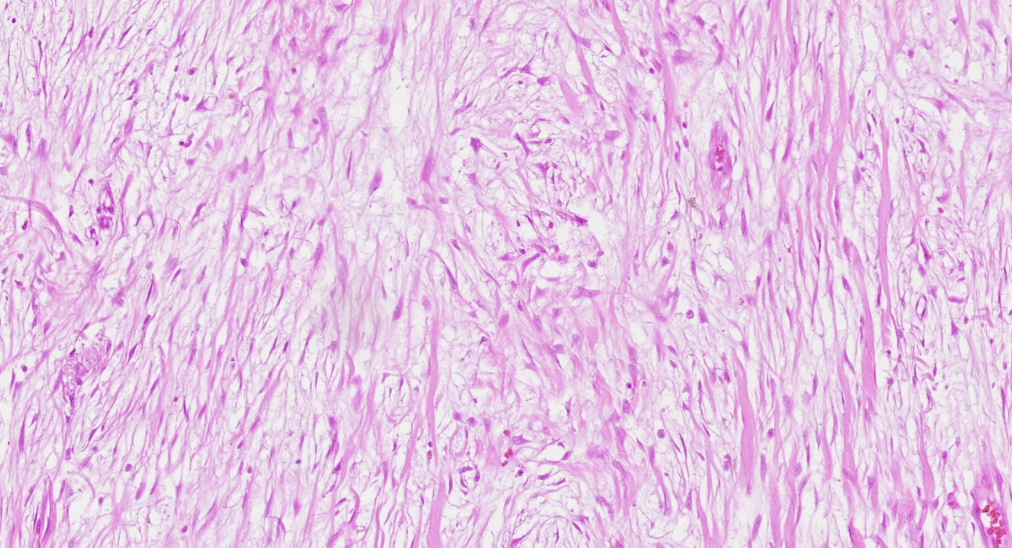
**

**Supplementary Figure 5. Histopathological features of the resected desmoplastic fibroma (H&E, ×40).** Final histopathological evaluation demonstrating uniform spindle-shaped fibroblasts arranged in interlacing fascicles within a densely collagenous stroma. The cells show minimal cytologic atypia, low mitotic activity, and no necrosis, all characteristic of desmoplastic fibroma. These findings confirmed the diagnosis on permanent section analysis.

**Supplementary Figure 6. OPG 15 months post-operation and reconstruction .** Panoramic radiograph obtained at 15-month follow-up demonstrating stable integration of the fibula graft with satisfactory bony continuity and preservation of mandibular contour. Imaging also shows appropriate growth of the right mandibular ramus with elongation of the reconstructed condylar complex, indicating preservation of the right condylar growth center and restoration of a functional condylar–ramus unit.

**Supplementary Figure 7. 15 months post-operation and reconstruction clinical presentation of the patient.** Extraoral and intraoral clinical photographs at follow-up demonstrating satisfactory facial symmetry and mandibular contour (left), with intraoral views showing healthy mucosa, stable occlusion, and preserved oral function (right).
